# Amygdala response predicts clinical symptom reduction in patients with borderline personality disorder: A pilot fMRI study

**DOI:** 10.3389/fnbeh.2022.938403

**Published:** 2022-08-30

**Authors:** Dirk E. M. Geurts, Thom J. Van den Heuvel, Quentin J. M. Huys, Robbert J. Verkes, Roshan Cools

**Affiliations:** ^1^Centre for Cognitive Neuroimaging, Donders Institute for Brain, Cognition and Behavior, Radboud University, Nijmegen, Netherlands; ^2^Department of Psychiatry, Radboud University Medical Center, Nijmegen, Netherlands; ^3^Department of Scelta, Expert Centre for Personality Disorders, GGNet, Nijmegen, Netherlands; ^4^Mental Health Neuroscience Department, Division of Psychiatry and Max Planck UCL Centre for Computational Psychiatry and Ageing Research, Institute of Neurology, University College London, London, United Kingdom; ^5^Kairos Center for Forensic Psychiatry, Pro Persona Mental Health, Nijmegen, Netherlands

**Keywords:** borderline personality disorder, dialectical behavior therapy (DBT), fMRI, amygdala, Pavlovian-to-instrumental transfer

## Abstract

Borderline personality disorder (BPD) is a prevalent, devastating, and heterogeneous psychiatric disorder. Treatment success is highly variable within this patient group. A cognitive neuroscientific approach to BPD might contribute to precision psychiatry by identifying neurocognitive factors that predict who will benefit from a specific treatment. Here, we build on observations that BPD is accompanied by the enhanced impact of the aversive effect on behavior and abnormal neural signaling in the amygdala. We assessed whether BPD is accompanied by abnormal aversive regulation of instrumental behavior and associated neural signaling, in a manner that is predictive of symptom reduction after therapy. We tested a clinical sample of 15 female patients with BPD, awaiting dialectical behavior therapy (DBT), and 16 matched healthy controls using fMRI and an aversive Pavlovian-to-instrumental transfer (PIT) task that assesses how instrumental behaviors are influenced by aversive Pavlovian stimuli. Patients were assessed 1 year after the start of DBT to quantify changes in BPD symptom severity. At baseline, behavioral aversive PIT and associated neural signaling did not differ between groups. However, the BOLD signal in the amygdala measured during aversive PIT was associated with symptom reduction at 1-year follow-up: higher PIT-related aversive amygdala signaling before treatment was associated with reduced clinical improvement at follow-up. Thus, within the evaluated group of BPD patients, the BOLD signal in the amygdala before treatment was related to clinical symptom reduction 1 year after the start of treatment. The results suggest that less PIT-related responsiveness of the amygdala increases the chances of treatment success. We note that the relatively small sample size is a limitation of this study and that replication is warranted.

## Introduction

Borderline personality disorder (BPD) is a prevalent and devastating psychiatric disorder associated with severe functional impairments and high mortality rates (American Psychiatric Association, 2000; Grant et al., 2008; Bolton and Robinson, 2010). Costs for society are high due to heavy use of expensive health care resources and persistent lack of productivity (Wunsch et al., 2014). Optimizing care for this patient group is of major importance (Gunderson, 2009).

Although several psychotherapeutic treatments exist for BPD, the response is highly variable and treatment effects are modest overall (Stoffers et al., 2012). For example, 27-35% of patients continue to have admissions, harm themselves, and conduct suicidal gestures (Lana and Fernández-San Martín, 2013). Only a few general predictors of outcome have been reported (Barnicot et al., 2012). The discovery of new outcome predictors is essential for the advancement of the field of personalized psychiatry. Neurocognitive mechanistic research might identify key predictors of available treatment outcomes and thus mitigate the large variability in treatment efficacy (Jones et al., 2015; Heinz et al., 2016; Huys et al., 2021). We report a proof-of-principle, pilot study focused on the relation between BPD symptom reduction over 1 year and affect-related neural processing, measured prior to the start of 1 year of dialectical behavior therapy (DBT).

Maladaptive and inflexible behavior in BPD has been argued to reflect derailed interaction between two principle controllers of human behavior, i.e., an instrumental and a Pavlovian controller (Hallquist et al., 2018). Instrumental control allows us to flexibly optimize our chances to achieve specific goals by learning what to do when (based on stimulus–action–outcome learning or operant conditioning). The Pavlovian system regulates inflexible, “automatic,” motivational responses in reaction to external and internal emotional stimuli (based on stimulus–outcome learning or classical conditioning). In the context of BPD, the interaction between Pavlovian and instrumental control, the so-called Pavlovian-instrumental transfer (PIT), is particularly worth investigating, as dysregulation of this interaction has been related to heightened impulsivity (e.g., behavioral activation instead of inhibition by aversive contextual cues) (Breland and Breland, 1961; Guitart-Masip et al., 2014; Hinojosa-Aguayo and González, 2020; Geurts et al., 2022), increased influence of emotional/motivational states (e.g., hampering effective goal pursuit) (Dolan and Dayan, 2013; Watson et al., 2014) and interpersonal hypersensitivity (cf. Hallquist et al., 2018), and a combination of symptomatology lying at the core of BPD (Gunderson, 2009). Here, we will probe whether BPD is indeed characterized by an aberrant influence of the Pavlovian system by assessing PIT in BPD patients and healthy controls. Critically, we will explore within the group of BPD patients whether neurocognitive correlates of PIT are related to symptom reduction over 1 year of DBT.

Relying on the biosocial model of emotion regulation, DBT is one of the leading evidence-based psychotherapies for BPD with the main focus on skillfully regulating impulsive and emotion-driven behavior (Linehan, 1993). DBT teaches how aversive motivational tendencies can be accepted and dealt with skillfully through the training of skills like mindfulness, distress tolerance, emotion regulation, and interpersonal effectiveness. Thus, DBT might help optimize the interaction between aversive motivational (Pavlovian) influences and instrumental behavior. In this manuscript, we assess the ‘vulnerability’ of instrumental, goal-appropriate behaviors to disruptions by aversive Pavlovian conditioned stimuli (CS). For this purpose, we used a previously validated behavioral PIT task that allows us to quantify the impact of motivational cues on instrumental decision-making.

Specifically, we measure aversive PIT, which refers to the observation that aversive instrumental actions, such as inhibition and withdrawal, are potentiated in the context of aversive Pavlovian CS, i.e., stimuli that predict aversive outcomes. Thus, aversive Pavlovian CS have been shown to inhibit instrumental approach actions (i.e., aversive inhibition) and to enhance instrumental withdrawal actions (Huys et al., 2011; Geurts et al., 2013a). Accumulating evidence from experimental studies with animals and healthy humans (Talmi et al., 2008; Prevost et al., 2012; Geurts et al., 2013a) and patients (Garbusow et al., 2016; van Timmeren et al., 2020) demonstrates the involvement of (prefrontal) limbic circuitry in PIT, including the ventral striatum and amygdala (Cardinal et al., 2002; Talmi et al., 2008; Balleine and Doherty, 2009; Prevost et al., 2012; Geurts et al., 2013a; Ly et al., 2014). The involvement of the amygdala is particularly relevant in the context of the current study, because the amygdala has also been central to neurocognitive theories and empirical research on BPD (Minzenberg et al., 2007; Hazlett et al., 2012; Soloff et al., 2017; Degasperi et al., 2021). For example, a recent meta-analysis reported functional hyperactivity of the left amygdala during aversive vs. neutral stimuli, as well as smaller gray matter volume of the amygdala in BPD (Schulze et al., 2016, 2019). This amygdala hyperactivation has been proposed to reflect the deviant salience of negative emotional stimuli and to be remediated by psychotropic medication (Schulze et al., 2016) and psychotherapy (Iskric and Barkley-Levenson, 2021) in BPD. We note that it is unclear whether remediation of amygdala hyperactivity is related to specific treatments or whether it is a general prerequisite for recovery from borderline symptomatology. Notwithstanding this ambiguity, evidence shows that effects of DBT are also associated with changes in blood oxygen level-dependent (BOLD) signal in the amygdala (Schnell and Herpertz, 2007; Krause-Utz et al., 2014; Salvador et al., 2016; Iskric and Barkley-Levenson, 2021). Here, we build on these previous findings by assessing the hypothesis that BPD is accompanied by abnormalities in aversive PIT and associated BOLD signal in the amygdala. Moreover, we ask whether aversive PIT and related amygdala signal before the start of therapy is associated with symptom reduction after treatment (Schmitt et al., 2016; cf. Schmitgen et al., 2019).

Thus, we hypothesize that borderline symptomatology might result from an imbalance between two major control systems of behavior: the motivational, reactive Pavlovian system on the one hand and a goal-oriented, instrumental system on the other. We explore this hypothesis by first investigating differences in baseline performance on a behavioral PIT task between healthy controls and BPD patients. Based on the above findings, we hypothesized that, relative to controls, BPD patients exhibit the enhanced impact of aversive Pavlovian CS on instrumental behavior, that is, greater aversive PIT (i.e., increased behavioral inhibition and withdrawal). Furthermore, we expect increased PIT-related BOLD signal in BPD relative to controls in the amygdala. Critically, we expect that the between-subject differences in amygdala response are related to symptom reduction across 1 year of DBT in the BPD group.

## Materials and methods

### Participants

To maximize external validity, we aimed for a patient sample that would represent patients treated in general mental health practice as closely as possible (Hoertel et al., 2015). Therefore, all patients who were enrolled in the pre-treatment phase of a 1-year DBT program at the Radboud University Medical Centre between March 2012 and March 2013 (*n* = 29) were invited to participate in this study. Twenty-three patients volunteered. Imaging datasets were obtained for 15 patients (all women), and clinical outcome measures after treatment were obtained for 14 of these patients (see Supplementary materials for details on inclusion). In addition, 16 healthy (MINI-plus) controls matched for gender and age were recruited per advertisement (for group demographics and questionnaire scores, see Table 1, and for comorbidity and medication use of the BPD group, see Supplementary Table 1). The local Medical Ethical Committee approved the study (NL36001.091.11), and consent was obtained from all participants.

**TABLE 1:** Demographical and clinical characteristics of the borderline personality disorder and healthy matched control participants.

	Healthy controls		Borderline personality disorder group			
	Baseline				1-year follow-up	
Size of group	*N* = 16		*N* = 15		*N* = 14	
	Mean	SD	Mean	SD	Mean	SD
Age	29.5	8.8	28.5	8.8	–	–
IQ (NLV)	101.8	12.3	100.3	11.5	–	–
Right handedness	16	–	14		13	
BPD47	6.7	6.5	79.7***	33.2	64.8^#^	30.0
OQ – total Sympt. distr. Inter. pers. Social role	42.5 19.1 8.8 8.8	20.6 10.6 4.9 4.2	91.5** 56.7** 20.2** 15.0**	19.2 14.1 3.8 4.5	79.1^##^ 50.0^#^ 17.5 11.6^#^	22.5 16.5 5.4 3.6
BDI-II	3.6	4.0	33.4***	14.3	28.4	14.0
BIS	18.4	7.1	23.5***	4.1	24.4	3.8
BAS	38.7	14.7	40.1***	5.8	41.4	5.1
Box Completion (s)	85.4	30.7	107.0**	20.9	96.8	27.4
Digit Span Forward Backward	13.2 7.1 6.0	2.5 1.6 1.3	16.2 8.3 7.9	4.0 1.9 2.4	15.2 7.7 7.5	4.1 2.2 2.4
Verbal Fluency	44.6	12.1	38.1	11.4	42.3	10.3

SD, standard deviation; NLV, Dutch reading test; BPD47, Borderline personality disorder checklist; OQ, outcome questionnaire; BDI-II, Beck depression index 2nd version; BIS, behavioral inhibition systems; BAS, behavioral activation system.

* Indicate significant differences between the groups (HC vs. BPD: *p < 0.05, **p < 0.01, ***p < 0.001)), # indciate significant differences between baseline and follow-up measurement (^#^p < 0.05, ^##^p < 0.01).

### Procedure

All patients enrolled in the pre-treatment phase of DBT were invited to attend three sessions: the first was a screening session, the second was a pre-treatment scan session just before treatment, and the third was a post-treatment assessment.

### Screening session

During the screening session, participants received a full diagnostic structured interview, which included the MINI-plus international neuropsychiatric interview and the Structured Clinical Interview for DSM-IV Axis II disorders (SCID-II), administered by a senior resident in psychiatry (author DG). To familiarize subjects during the first visit with the scanning environment and procedures, we employed a short scan session of about 15 min during which a structural MRI scan was obtained and subjects were familiarized with the instructions and instrumental and Pavlovian training stages in the scanner.

### Pre-treatment scan session

**FIGURE 1:**
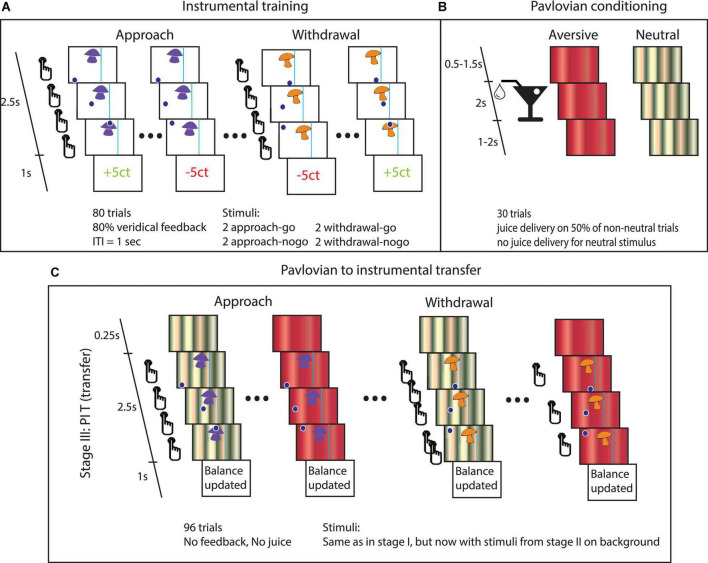
Task details. **(A)** Instrumental stage. Trials started with the appearance of the instrumental stimulus at the top center of the screen and a dot at the bottom of the screen. In approach trials, the dot appeared either on the left or on the right bottom of the screen. From left to right: Participants could choose to do nothing (approach-no-go), in which case the dot would move past the instrumental stimulus. Alternatively, they could press the button repeatedly to steer the dot through the instrumental stimulus (approach-go). In withdrawal trials, the dot started centrally beneath the instrumental stimulus. Participants could choose to press the button repeatedly to avoid moving through instrumental stimulus (withdrawal-go) or to do nothing (withdrawal-no-go). If the dot entered the target region, then the instrumental stimulus was ‘collected’. The vertical line to one side of the instrumental stimulus could not be crossed by the dot. **(B)** Pavlovian conditioning. Participants were presented with different stimuli that were followed by juice delivery. **(C)** PIT stage. The PIT stage paralleled the instrumental training, except that Pavlovian CS tiled the background. The effect of interest is how the Pavlovian CS changed instrumental behavior (mean proportion of go-actions and the average number of button presses over the go-actions). Note that the trials involving the appetitive CS were omitted from this figure, because this particular paradigm has been shown to be insensitive to detecting appetitive PIT (see Supplementary material) and our hypotheses concern aversive PIT.

During the second visit, before treatment started, subjects completed several questionnaires (Table 1), of which the Borderline Personality Disorder Checklist (BPD47) measuring the symptom severity was of primary interest. Before entering the scanner, instructions on the computer task were repeated orally. After receiving the instructions for a third time, now projected on the scanner screen, they started the PIT paradigm (Figure 1). After a 15-min break, subjects performed a short neuropsychological test battery (Table 1).

### Treatment

Participants received a 1-year group version of the standard DBT protocol (Linehan, 1993; Gutteling et al., 2012) divided into the standard 4 weekly components (DBT group psychotherapy, groups skills training, 24/7 telephone coaching, and a therapist consultation team). The program differed from standard DBT only in that the weekly psychotherapy sessions were offered not individually but in groups. All DBT strategies (dialectics, behavior chain analysis, radical acceptance strategies of validation and mindfulness, contingency management, exposure, cognitive restructuring, and skills training) were used across all components addressing the five functions of DBT (increasing behavioral capabilities, improving motivation for skillful behavior, generalization of skills to the natural environment, reinforcement of functional over dysfunctional behavior, and enhancing therapist effectiveness) and were performed by well-trained DBT therapists and skill trainers. Although more elaborate research is needed to show that scaled versions as described above are as effective as standard DBT, Gutteling et al. (2012) demonstrated evidence that suggests that this scaled version of DBT is as effective as standard DBT for the treatment of borderline patients.

### Post-treatment, follow-up session

The third and final follow-up session followed after treatment had ended, approximately 1 year after the pre-training scan session. Subjects completed the same questionnaires and participated in the same neuropsychological test battery as in the second session (Table 1). In addition, the MINI was administered once again to investigate whether axis I classifications had changed and the BPD47 to measure changes in borderline symptom severity.

### Pavlovian-instrumental transfer paradigm

Participants performed a computerized PIT task to assess how instrumental approach and withdrawal actions are influenced by aversive Pavlovian CS, i.e., aversive PIT (Geurts et al., 2013a). The experiment consisted of three stages: (1) instrumental conditioning, (2) Pavlovian conditioning, and (3) PIT (see Figure 1 for a global overview and Table 2 for details on the experimental layout).

**TABLE 2:** Experimental layout.

	# trials/time per trial(s)	# stimuli	Reinforcement
Instrumental training	80/2.5s		
Blocks of 8 trials per Action Context (approach/withdrawal)	20	2 stimuli requiring approach: S^I^_1_,_2_	80% reward/20% punishment for go
	20	2 stimuli requiring approach-nogo: S^I^_3_,_4_	80% reward/20% punishment for nogo
	20	2 stimuli requiring withdrawal: S^I^_5_,_6_	80% reward/20% punishment for go
	20	2 stimuli requiring withdrawal-nogo: S^I^_7_,_8_	80% reward/20% punishment for nogo
Pavlovian training	60/3s	3	
Each 10^th^ trial a query trial to choose between two CS	20	1 stimulus followed by aversive juice → aversive CS: S^P^_1_	50% of trials are reinforced
	20	1 stimulus without reinforcement → neutral CS: S^P^_2_	No reinforcement
	20	1 stimulus followed by appetitive → juice appetitive CS: S^P^_3_	50% of trials are reinforced
Pavlovian to instrumental transfer	96/2.5s	4/3	
Blocks of 8 trials per Action Context (approach/withdrawal)	32	S^I^_1_,_2_,_3_,_4_,_5_,_6_,_7_,_8_ | S^P^_1_	No direct reinforcement
	32	S*^I^*_1_,_2_,_3_,_4_,_5_,_6_,_7_,_8_ | S*^P^*_2_	No direct reinforcement
	32	S*^I^*_1_,_2_,_3_,_4_,_5_,_6_,_7_,_8_ | S*^P^*_3_	No direct reinforcement
**2 Runs**			

*Stage 1.* Participants performed an instrumental learning task to earn as much money as possible. There were two Action Contexts in this task: (i) One in which the active response led to an approach and (ii) another in which the active response led to a withdrawal. In each context, different instrumental stimuli (mushrooms/shells) were repeatedly presented to the participant (Figure 1A). In the approach, Action Context participants learned through monetary feedback (wins and losses) whether to ‘collect’ the instrumental stimulus (approach-go) or not (approach-no-go). In the withdrawal Action Context, they learned to avoid collecting instrumental stimuli (withdrawal-go) or not (withdrawal-no-go).

In both the approach and withdrawal Action Contexts, there were two go-stimuli, which yielded reward more often (i.e., 80% of the cases) after active responses (and punishment after not responding), and two no-go-stimuli, which yielded reward more often (i.e., also 80% of the cases) after not responding (and punishment after go-responding). Instrumental learning was assessed by calculating the proportion of correct responses (p(correct)) over time.

*Stage 2.* In this Pavlovian stage, different Pavlovian CS were conditioned (Figure 1B). During a classical conditioning procedure, three audiovisual stimuli were presented. The appetitive and aversive conditioned stimuli (CS) were followed, respectively, by appetitive or aversive juice (i.e., the unconditioned stimuli, USs) on 50% of trials. The neutral CS resulted in no outcome. The appetitive juice was based on subjective preference for apple, orange, or strawberry lemonade. The aversive juice was a bitter magnesium sulfate solution (0.3M).

Conditioning was assessed in two ways: (1) participants indicated the degree to which they liked each of the CS (and USs) by using a visual analog scale (VAS), before and after the experiment; and (2) participants chose one of the two presented Pavlovian stimuli (presented for 2 s; ITI 0.5 s) in extinction on 12 interspersed query trials during the Pavlovian stage.

*Stage 3.* In the PIT stage, we tested how instrumental approach and withdrawal actions (trained in *stage 1*) are influenced by aversive Pavlovian CS (*conditioned in stage 2.)*. Therefore, stimulus presentation was the same as in the instrumental stage, except that Pavlovian CS from the Pavlovian stage 2 tiled the background from 250 ms before and during the trials, and this stage was run in nominal extinction, i.e., no juice or monetary outcomes were presented (Figure 1C). Participants were instructed that their choices counted toward the final monetary total and that the juices associated with the Pavlovian outcomes were collected outside the scanner for them to drink afterward. Whether instrumental approach and withdrawal actions were influenced by aversive Pavlovian CS was assessed per Action Context (approach/withdrawal) and CS stimulus (neutral/aversive).

There were two independent runs separated by a 2-min break (each including run-specific stimuli/CS), with each run including all three stages. Each instrumental stimulus was presented 12 times and each Pavlovian CS 32 times. These Pavlovian CS were counterbalanced over the eight instrumental stimuli.

### Image acquisition

Whole-brain imaging was performed on a 1.5 Tesla MR scanner (Avanto, Siemens Medical Systems, Erlangen, Germany). Functional data were obtained using a multi-echo gradient T2*-weighted echo-planar (ME-EPI) scanning sequence (Poser et al., 2006) (see Supplementary materials for details).

### Analysis

Our primary analysis was restricted to the PIT stage. Analysis and results of the instrumental and Pavlovian training data are presented in the Supplementary materials. The analyses presented below consist of two parts: First, we assessed the effects of the group on behavior and fMRI BOLD response during the PIT stage, measured at baseline. Here, we focus on both the behavioral and fMRI analyses in line with our hypothesis on aversive PIT. We discern two aspects of aversive PIT: Action Context-specific aversive PIT and aversive PIT that is independent of Action Context, i.e., aversive PIT across Action Contexts. Action Context-specific aversive PIT quantifies the differential effect of an aversive CS on approach and withdrawal behavior, whereas aversive PIT across Action Contexts quantifies general effects of the Pavlovian CS on instrumental behavior irrespective of whether it is approach or withdrawal behavior. Statistically, aversive PIT across Action Contexts is captured by the main effect of CS Valence (neutral vs. aversive across Action Contexts), while Action Context-specific aversive PIT is captured by the interaction between CS Valence and Action Context. These different aversive PIT effects have been associated in previous studies with different clinical outcomes and neural mechanisms (Geurts et al., 2013a,b; Garbusow et al., 2016; Huys et al., 2016). Specifically, while Action Context might arise from a vmPFC-dependent process (Geurts et al., 2013a) that is predictive of recovery from depression (Huys et al., 2016), the extent to which Pavlovian CS inhibit ongoing behavior across Action Contexts likely reflects amygdala/striatal activity and changes in serotonergic transmission (Geurts et al., 2013b), and is instead associated with the psychopathic tendency in a sample of violent offenders (unpublished findings, submitted to the current special issue of Frontiers in Behavioral Neuroscience).

Second, within the BPD group, we assessed whether aversive PIT and associated BOLD signals were associated with symptom reduction at the end of the 1-year DBT program.

We note in addition that our previous work in healthy controls, on which the current study builds, revealed that the current paradigm was not sensitive to (and therefore less valid to assess group effects on) appetitive PIT (Geurts et al., 2013a). We therefore only present the data on aversive PIT. In the Supplementary material, we confirm that, indeed, the current paradigm is not sensitive to appetitive PIT.

### Pavlovian-instrumental transfer

#### Behavioral analyses

We focused our analyses on aversive PIT, i.e., the effect of aversive Pavlovian CS on instrumental behavior. The effects of Action Context (approach/withdrawal), CS Valence (neutral/aversive), and group (healthy controls/BPD patients) in the critical transfer test were assessed in terms of proportion of go-choices [p(go)] and the average number of button presses (BP, made during these go-choices). Note that our previous work in healthy controls, on which the current study builds, revealed that the current paradigm was not sensitive to (and therefore less valid to assess group effects on) appetitive PIT (Geurts et al., 2013a). We present behavioral data on appetitive PIT in the Supplementary materials.

Thus, analyses were targeted at the degree to which aversive CS influenced instrumental behavior. More specifically, we analyzed across Action Context (approach and withdrawal) how much the aversive Pavlovian CS (compared with the neutral CS) inhibited instrumental ‘go’ responding (i.e., the main effect of CS Valence). In addition, we also assessed the Action Context specificity of aversive PIT, i.e., to what extent the effect of the aversive Pavlovian CS is dependent on Action Context (i.e., interaction CS Valence X Action Context). The dependent variables were first averaged across runs and normality was assessed, before they were submitted to a repeated measures ANOVA (rmANOVA), with Action Context (approach/withdrawal) and CS Valence (neutral/aversive) as within-subject factors and group (healthy controls/BPD patients) as a between-subject factor. Due to non-normal distribution of p(go), we employed non-parametric tests to assess whether there was a significant aversive PIT effect across groups (related-samples Wilcoxon signed-rank test comparing the difference between p(go) for neutral Valence and p(go) for aversive as a function of Action Context) and whether there was a difference in aversive PIT between groups (independent samples median test comparing the compound measure of Action Context-specific aversive PIT, i.e., [(approach neutral- approach aversive – (withdrawal neutral - withdrawal aversive)] and aversive PIT across Action Contexts [(approach neutral + withdrawal neutral – (approach aversive - withdrawal aversive)] between groups).

#### fMRI analysis

An fMRI analysis was performed with SPM5 software (Wellcome Trust Centre for Cognitive Neuroimaging, London, United Kingdom). Pre-processing steps and first-level fMRI analysis were identical to those employed by Geurts et al. (2013a): First, realignment parameters were estimated for the images acquired at the first echo time and consequently applied to images resulting from the three other echoes. The echo images were combined by applying a PAID-weight algorithm assessing the signal-to-noise ratio as described by Poser et al. (2006). Thirty volumes, acquired before each instrumental training session, were used as input for this algorithm. Thereafter, the following preprocessing steps were applied: slice-time correction, co-registration, and a segmentation procedure using the tissue probability maps provided by SPM5 for gray matter, white matter, and CSF centered in MNI space to estimate normalization parameters based on the structural image. Structural and functional images were then normalized by applying these estimations. All normalized images were smoothed with an isotropic 8 mm full-width half-maximum Gaussian kernel (Worsley and Friston, 1995). The fMRI analysis was restricted to the PIT stage and was similar to our previous analyses (Geurts et al., 2013a). The general linear model (GLM, Supplementary Figure 1) at the participant level consisted of six main regressors representing the onset of the six different PIT trials [Action Context (approach/withdrawal) x CS Valence (appetitive/neutral/aversive)]. For each main regressor, an additional parametric regressor was added (Büchel et al., 1996): The PIT regressor (Talmi et al., 2008; cf. Geurts et al., 2013a) was a parametric modulator of BOLD responses by the number of button presses per trial. Contrasting this regressor between the different CS Valence measures thus reveals “PIT-related regions”, i.e., regions where the BOLD signal is associated with valence-dependent coupling between amygdala BOLD signal and instrumental behavior on a trial by trial basis. Note that such a contrast goes beyond simple reactivity of a region to a CS or to instrumental behavior *per se*; it critically captures its interaction, i.e., PIT. A further parametric regressor contained the expectation associated with each instrumental stimulus (the *Q*-value) per trial as estimated from a model-based analysis of behavior (Huys et al., 2011) applied to the current data. This was done based on prior data showing that the BOLD signal in the prefrontal cortex and striatum, our regions of interest, covaries with instrumental action value (O’Doherty, 2004; Valentin et al., 2007; Wunderlich et al., 2009; Smith et al., 2010; Jocham et al., 2011; see for meta-analysis, Chase et al., 2015). As such, this approach maximized the degree to which our GLM captured variability in the relevant BOLD signals. Furthermore, realignment parameters were added, high-pass filtering (128s) was applied, and parameter estimates were obtained by maximum-likelihood estimation (AR1).

The parameter estimates for the neutral and aversive parametric PIT regressors were used in a 2 × 2 × 2 rmANOVA at the group level (with random effects) with Action Context (approach/withdrawal) and Valence (neutral/aversive) as within-participant factors and group (healthy controls/BPD) as a between-participants factor. Within this rmANOVA, we assessed Action Context-specific aversive PIT and aversive PIT across Action Context for group differences. Moreover, we also assessed the main effect of Action Context. Based on Geurts et al. (2013a), we expected this analysis to reveal that the BOLD signal in the ventromedial prefrontal cortex would be Action Context-specific (approach > withdrawal). We did not expect a group effect on this contrast.

**FIGURE 2:**
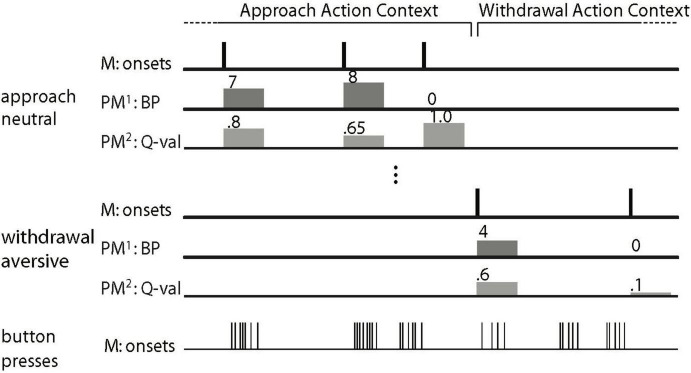
Schematic depiction of the general linear model to analyze the Pavlovian-instrumental transfer (PIT) data (Figure after Talmi et al., 2008; Geurts et al., 2013b). The main regressors (M) model the onset of a trial as a delta function. There is a main regressor for each of the six trial types. For all six main regressors, there are two parametric modulators (PM). The first parametric modulator (PM^1^), the PIT regressor, consists of the number of button presses made per trial (0 for no-go). In the 7th main regressor (of no interest), every single button press is modeled by a delta function. For reasons of clarity, only two of the six trial types (approach neutral and withdrawal aversive) are depicted. The regressors of no interest are not shown (i.e., the movement nuisance regressors and the second parametric modulator).

To capture additional PIT signals related to stable patterns of behavior beyond trial-by-trial variation in instrumental vigor, we contrasted the main regressors (Figure 2) at the participant level to calculate the main effect of Valence [(approach&neutral + withdrawal&neutral) - (approach&aversive + withdrawal&aversive)] (cf. Talmi et al., 2008; cf. Geurts et al., 2013a). The resulting SPM was then used in a two-sample *t*-test at the group level with aversive PIT in terms of the average number of button presses as a covariate for each group separately enabling comparison between groups. Based on Geurts et al. (2013a), we expected that behavioral aversive PIT across Action Contexts in terms of the average number of button presses [(BP| approach&neutral + BP| withdrawal&neutral) - (BP| approach&aversive + BP| withdrawal&aversive)] would be related to BOLD signal change (neutral-aversive) in the amygdala and nucleus accumbens and that this relationship would differ between the groups (i.e., a stronger correlation within the BPD group).

#### Treatment success and its prediction

Our primary measure of treatment success was the Borderline Personality Disorder Checklist, (BPD47, Bloo et al., 2017) a 47-item self-report questionnaire based on the Borderline Personality Disorder Severity Index (Arntz et al., 2003). Furthermore, as secondary measures, we also assessed the quality of life with the Outcome Questionnaire (OQ, Lambert et al., 1996) and depressive symptoms with the Beck Depression Inventory second edition (BDI-II, Beck et al., 1996). The treatment effect was computed by subtracting the post-treatment scores from those acquired during the first scan session.

#### Predictive relationship between aversive Pavlovian-to-instrumental transfer and symptom reduction

We assessed the association between aversive PIT and associated BOLD signal [at the whole-brain level and within the predefined amygdala region of interest (ROI)], measured pre-treatment, with clinical symptom reduction 1 year later. A second-level random-effects simple regression analysis was conducted to assess whether PIT-related neural signal was associated with symptom severity at baseline, and/or symptom reduction over 1 year. To this end, we computed Action Context-specific aversive PIT-related BOLD signal [(PITregressor| approach&neutral - PITregressor| approach&aversive) - (PITregressor| withdrawal&neutral - PITregressor| withdrawal&aversive)], aversive PIT-related BOLD signal across Action Contexts [PIT regressor| approach&neutral + PIT regressor| withdrawal&neutral – (PIT regressor| approach&aversive + PIT regressor| withdrawal&aversive)], as well as BPD47 scores at baseline and BPD47 change (before-after). These latter covariates of interest were tested in two simple regression analyses of the aversive PIT statistical parametric maps. Any relationship between the PIT-related BOLD contrasts and the BPD47 change (without a baseline relationship) would indicate that PIT-related signaling is predictive of symptom reduction. In addition, as a sensitivity analysis because of the small sample size, we also performed the non-parametric equivalent of this analysis with SnPM (Winkler et al., 2014) and we employed a leave-one-participant-out procedure (Esterman et al., 2010), in which a single participant is iteratively left out of the second-level correlational analysis. The resulting clusters within the anatomically defined bilateral amygdala (thresholded at *p* < 0.001 uncorrected) were then used to extract the mean beta weights of the left-out participant to calculate the aversive PIT contrast. This procedure was repeated for each participant. The GLM from the remaining participants thus serves as an independent localizer for the participant left out (Esterman et al., 2010).

#### Statistical thresholding

We report effects that survive family-wise error (FWE) correction for multiple comparisons across the whole brain (P_WB_ < 0.05, voxel-level) or in one of the following ROIs: The amygdala (automated anatomical labeling atlas, Tzourio-Mazoyer et al., 2002) was our primary ROI to assess the effect of symptom reduction. Both the amygdala and nucleus accumbens (same as in Geurts et al., 2013a) were chosen as ROIs for the analysis of the main PIT task effects (across and between groups) based on their key role in PIT (Corbit, 2005; Talmi et al., 2008; Corbit and Balleine, 2011; Prevost et al., 2012; Geurts et al., 2013a; Garbusow et al., 2016). Specifically, in our previous study, we found BOLD response in both these regions to be associated with behavioral PIT on a participant-by-participant basis. Following our prior work, we also assessed action specificity in the ventromedial prefrontal cortex: The region shown to be sensitive to Action Context in our previous PIT study was used as ROI (MNI coordinates of ROI center: xyz = [-8 36-8]) (Geurts et al., 2013a). The left and right elements of each bilateral volume of interest were combined using Marsbar™ (Brett et al., 2002).

## Results

### Baseline behavioral data

#### Pavlovian-instrumental transfer

Consistent with our previous studies using this paradigm, we observed opposite effects of the aversive Pavlovian CS on approach and withdrawal actions (in terms of choice p(go), Figure 2): aversive Pavlovian CS inhibited approach and activated withdrawal actions. Planned contrasts confirmed the statistical significance of this action specificity of the aversive PIT effect (related-samples Wilcoxon signed rank test [p(go| approach&neutral) - p(go| approach&aversive)] > [p(go| withdrawal&neutral)- p(go| withdrawal&aversive)]: p = 0.031, one-tailed). There were no differences between the groups (independent samples median test: *p* = 0.48), but we note that the action-specific PIT effect was present in healthy controls (*p* = 0.008), but not in patients (*p* = 0.860) when examined separately.

There were no main task effects except for the main effect of Action Context in terms of the average number of button presses (F_(1_,_29)_ = 33.7, *p* < 0.001, all other *F* < 1.8 and *p* > 0.2, Supplementary Table 2). There were no group differences.

Performance on the instrumental task and assessments of Pavlovian training also did not differ between the groups (Supplementary Results). To be complete, we confirmed the already established insensitivity to detect appetitive PIT with the current paradigm (Supplementary Results).

#### Baseline imaging data

**FIGURE 3:**
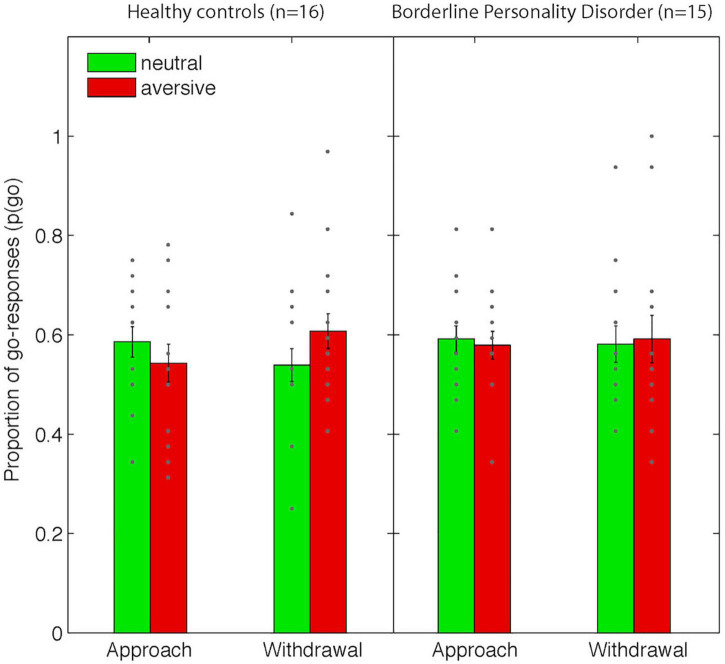
Behavioral data from the Pavlovian-instrumental transfer stage. Shown are mean proportions of go-responses [p(go)] as a function of Action Context (approach vs. withdrawal) and Valence (neutral/aversive). Error bars represent standard errors of the means and dots represent individual data points. Note that there were no significant differences between groups.

Consistent with our previous fMRI study using this paradigm, trial-by-trial instrumental action-related BOLD signal in the vmPFC varied as a function of Action Context. The BOLD signal was greater during approach than during withdrawal (small volume corrected results for the vmPFC ROI: peak voxel MNI-coordinates [−6 32 −12], *k* = 45, *Z* = 3.86, p_FWE_ = 0.021, Figure 3).

Conversely, we did not replicate the previously observed correlation between individual differences in behavioral aversive PIT and BOLD signals in the amygdala and nucleus accumbens. Moreover, we did not find significant main effects of or interactions with the factor group.

#### Aversive Pavlovian-to-instrumental transfer and symptom reduction

##### Symptom reduction

The 14 patients who were seen at follow-up, 1 year after the start of therapy, showed a significant reduction in symptom severity as measured with the BPD47 (mean difference = −17.3, t_13_ = 2.5, *p* = 0.027, reliable change index (Jacobson and Truax, 1991): 15.8), OQ (mean difference = −12.4, t_13_ = 3.1, *p* = 0.009), and in trend with the BDI-II (mean difference = −4.8, t_13_ = 1.8, *p* = 0.090).

None of the neuropsychological tests reported in Table 1 changed significantly from baseline to 1 year after treatment (all −1.9 > t_13_ < 2.2, all *p* ≥ 0.05).

##### Pavlovian-to-instrumental transfer-related BOLD signal in the amygdala is related to symptom reduction 1 year later

**FIGURE 4:**
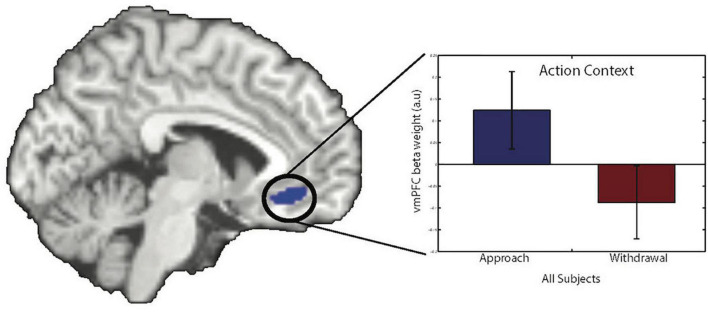
Action-specific BOLD response in the vmPFC. There was a main effect of Action Context in the vmPFC (peak voxel MNI-coordinates [–6 32 –12], *k* = 45, *Z* = 3.86, p_FWE_ = 0.021, small volume corrected). The bar graph shows parameter estimates from the peak voxel for the different Action Contexts (error bars show SEM). Images are displayed at a statistical threshold of *p* < 0.001 uncorrected.

Pre-treatment PIT-related BOLD signal in the bilateral amygdala was related to BPD symptom reduction after 1 year (Figure 4). Higher aversive PIT-related signals across Action Contexts were associated with less symptom reduction 1 year later. This observation was substantiated by using both parametric and non-parametric statistical analyses (small volume corrected effects in the amygdala; parametric tests with SPM: peak voxel MNI-coordinates [−24 0 −16], *k* = 22, *Z* = 3.79, p_FWE_ = 0.027; non-parametric test with SnPM: peak voxel MNI-coordinates [−24 0 −18], pseudo-t = 4.22, p_FWE_ = 0.013; and MNI-coordinates [22 4 −18], pseudo *t* = 3.19, p_FWE_ = 0.06). The robustness of these effects was confirmed by cross-validation (r_(14)_: −0.655, *p* = 0.011) and by supplementary analyses on mean beta estimates extracted from the anatomically defined bilateral amygdala (Pearson r_(14)_: −0.667, *p* = 0.009). Note, that no significant relation was observed between baseline BPD47 scores and PIT-related amygdala signal (Pearson r_(14)_:0.33, *p* = 0.25).

Next, we explored the specificity of this predictive effect with respect to other (more easily acquired) baseline measures, including baseline BPD47, OQ, BDI-II, BIS, BAS, box completion time, verbal fluency, and digit span (Table 1). A stepwise linear regression analysis (with criteria probability of *F* to enter ≤ 0.05 and o F to remove ≥ 0.10) identified two predictors of symptom reduction. Indeed, pre-treatment PIT-related signal in the bilateral amygdala accounted for variance in symptom reduction over and above the other collected baseline measures. Verbal fluency was the only other selected predictor of symptom reduction (Final regression model including PIT-related amygdala signal and verbal fluency: F_(2_,_13)_ = 11.6, *p* = 0.002, standardized coefficients beta for amygdala signal:0.64, *p* = 0.003; and for the verbal fluency: −0.48, *p* = 0.016). All other measures did not enter the model (all | t| <1.5, all *p* > 0.2). Next, we examined whether the predictive effect of pre-treatment PIT-related amygdala signal was specific to BPD47 change or whether it extended to other changes in clinical or neuropsychological measures. Indeed, stepwise multiple regression analysis with this amygdala signal as a dependent variable revealed that the association of this signal with BPD47 improvement (*F*_(1_,_12)_ = 9.6, *p* = 0.009) did not extend to any of the other changes in clinical or neuropsychological measures (all | t| < 1.9, all *p* > 0.18). This is relevant because improvement in borderline severity was accompanied by improvement in depressive symptoms as measured with the BDI-II (r_14_ = −0.67, *p* = 0.008), as well as improvement in verbal fluency (r_14_ = 0.91, *p* < 0.001).

## Discussion

Results failed to confirm our prediction that patients with borderline personality disorder exhibit abnormal aversive PIT compared to healthy controls at the group level. However, on an individual level, the results demonstrate that the BOLD signal in the amygdala elicited during the aversive PIT task is related to symptom reduction in these patients across 1 year of follow-up. Greater PIT-related responsiveness of the (bilateral) amygdala was associated with reduced clinical improvement 1 year later. More specifically, this suggests that individual differences in the degree to which amygdala processing relates to trial-by-trial instrumental responding in the context of an aversive Pavlovian CS predict resistance to clinical improvement of (or slower recovery from) BPD. Thus, participants who showed increased coupling between the amygdala BOLD signal and instrumental behavior during aversive Pavlovian CS presentation showed less clinical improvement. In more general terms, this suggests that individual differences in amygdala response could predict clinical improvement of BPD.

Based on observations that BPD is associated with the abnormal impact of aversive stimuli on behavior (Soloff et al., 2017; Hallquist et al., 2018), we employed an aversive PIT task that measures the degree to which aversive Pavlovian CS alter instrumental behavior. We replicated the previously observed basic behavioral task effects, including the Action Context-specificity of aversive PIT (Huys et al., 2011; Geurts et al., 2013a), with an aversive Pavlovian CS suppressing approach, but potentiating withdrawal actions. These task effects were not modulated by BPD, although when analyzing the groups separately, we only found significant effects in the healthy controls. The absence of a group effect might be due to the relatively stressful scanner environment (Talmi et al., 2008; cf. discussion of Geurts et al., 2013a). Indeed, there are indications that stress reduces behavioral PIT effects (Quail et al., 2016; but see Pool et al., 2015) and patients with BPD might be more sensitive to this stress. It might also be a consequence of the use of psychotropic medication in about two-thirds of our patients, which has been associated with attenuated amygdalar hyperreactivity in BPD (Schulze et al., 2016) and is likely to change PIT through changing monoaminergic signaling (cf. Geurts et al., 2013b; Hebart and Gläscher, 2015; Swart et al., 2017). Moreover, given the small sample sizes, the absence of a group effect on action-specific PIT might also reflect insufficient statistical power to detect such a difference. However, we cannot exclude that, as a group, BPD patients indeed do not exhibit abnormal aversive PIT.

The key observation of this study is that neural activity of the amygdala in BPD patients is associated with clinical symptom reduction. These results substantiate the promise of neurocognitive strategies for predicting treatment outcomes in various psychiatric disorders (Nitschke et al., 2009; Pizzagalli, 2010; Roiser et al., 2011; Månsson et al., 2015; Perez et al., 2016; Garbusow et al., 2016; Huys et al., 2016; Schmitgen et al., 2019; Westlund Schreiner et al., 2019; Sampedro et al., 2021). The considerable gap between cognitive neuroscience and clinical practice has been the subject of a fruitful ongoing debate (Paulus et al., 2016; Stephan et al., 2016; Huys, 2018). One major problem in the clinical relevance of neurocognitive research is that most studies have compared groups of patients, failing to address individual differences in the treatment efficacy. Future work is required to investigate whether an aversive PIT-related neural signal is associated selectively with DBT efficacy, or rather reflects general treatment efficacy or even BPD symptom change more irrespective of treatment.

Moreover, our results provide converging evidence for the validity of the PIT paradigm for predicting clinical symptom changes [in depression (Huys et al., 2016) and addiction (Garbusow et al., 2016)]. It should be noted that, here, amygdala signal across Action Contexts was the predictor, whereas in the study of Huys et al. (2016), it was the Action Context specificity of behavior that predicted recovery from depression. We did not find such an association for symptom reduction in patients with borderline personality disorder. Moreover, in the study of Garbusow et al. (2016), it was the PIT effect in the nucleus accumbens that predicted relapse in alcohol use. This suggests that different aspects of the neurocognitive mechanisms underpinning the transfer between Pavlovian CS and instrumental behavior might be disorder and/or treatment specific. We note that these studies, just like the current study, are relatively small in sample size. Nevertheless, these studies make concrete steps in translating hypotheses on mechanistic relevance for clinical treatments and as such are stepping stones for larger future studies making use of their methodology, which are already emerging (e.g., Chen et al., 2021).

The present results suggest that symptom reduction after DBT is greater in BPD patients who show lower amygdala signals during aversive PIT. The finding that the amygdala signal is predictive of symptom reduction in BPD after DBT concurs with empirical findings and neurocognitive theories, implicating a central role for the amygdala in BPD (Schulze et al., 2016, 2019) and DBT (Schnell and Herpertz, 2007; Goodman et al., 2014; Schmitt et al., 2016; Schmitgen et al., 2019). Several recent studies have shown changes in amygdala signaling after DBT (Schnell and Herpertz, 2007; Goodman et al., 2014; Schmitt et al., 2016; Niedtfeld et al., 2017; but see Winter et al., 2017). Schnell et al. employed a pilot study with six BPD patients who received several fMRI scans during 3 months of DBT. The four patients who responded to DBT all showed decreases in amygdala BOLD responses to emotional pictures. In keeping with this finding, Goodman et al. (2014) reported decreases in amygdala responses to emotional pictures and associated improvement in self-reported emotional regulation in 11 BPD patients after 1 year of DBT treatment. Moreover, Niedtfeld et al. (2017) showed in 28 patients with BPD that a scaled version of 12 weeks of DBT attenuated amygdala deactivation in response to pain. Schmitt et al. (2016) showed that patients who responded well to DBT exhibited reduced activation in, among other regions, the amygdala, during the reappraisal of negative stimuli after DBT.

These studies suggest that the association between amygdala signaling and symptom reduction, observed in the current study, might relate to treatment-induced changes in the amygdala. We stress, however, that we did not collect behavioral or fMRI data after therapy, which precludes us from assessing whether the amygdala signal indeed changed during this treatment, or whether it is a stable trait that indexes the susceptibility to the offered treatment. Moreover, due to the absence of a control condition in our design, we restrict our conclusions to the general case of clinical improvement. Thus, we cannot claim the specificity of our results to DBT. Moreover, PIT-related amygdala signal might also reflect more general, less treatment-specific, process underlying improvement like the ability to (emotionally) engage and/or commit oneself to treatment. We thus restrict our conclusion to the general *predictive* effect of amygdala signal on symptom change.

Although several studies, as mentioned above, assessed pre- to post-therapy changes in neural processing in BPD, so far, only two other studies assessed the value of selectively pre-treatment task-based fMRI signals for predicting treatment success (Perez et al., 2016; Schmitgen et al., 2019). In the study by Perez et al. (2016) including 10 patients with BPD, a greater pre-treatment BOLD signal in the right anterior cingulate cortex during an emotional go/no-go task was associated with reduced improvement after transference-focused psychotherapy (TFP) in terms of the factor ‘constraint’ of the multidimensional Personality Questionnaire. Moreover, a greater BOLD signal in the left posterior-medial OFC/ventral striatum was associated with reduced improvement in terms of the total score on the Affective Lability Scale. The study of Schmitgen et al. (2019) is of specific interest for the current study, because it explicitly addressed the prediction of clinical DBT effects based on, amongst others, task-based fMRI in a relatively large sample (n = 31) of BPD patients with a sophisticated cross-validation procedure to optimize a random forest prediction algorithm. They employed three emotion regulation tasks, fMRI, and structural MRI before 12 weeks of DBT. They showed that (left) amygdala (and parahippocampus) activation during a cognitive reappraisal task was particularly informative for treatment response prediction. Accuracy of predicting treatment response (base rate 52%) of the model based on solely these fMRI data reached 75%. Of note is that responders, while instructed to look at negative emotional pictures, showed lower left amygdala reactivity before therapy compared to non-responders. Together with these prior data, our findings strengthen the observation that particular limbic circuitry processing during affective action regulation renders BPD patients more resistant to clinical improvement after therapy. Moreover, differences between these studies employing two different treatment regimes (TFP vs. DBT) might speak to the future practical, clinical use of these findings. Future research should investigate how we can make treatment regimes more efficient by allocating specific patients to specific treatment modalities based on their functional neural signature. Thus, combining different neural predictors for treatment success specific to different treatment modalities might help us to reveal which patients should be allocated to which treatment. Before being able to implement this in clinical practice, more large-scale practice-based studies should be carried out to ensure the reliability and clinical usefulness of these predictions. Our data provide proof of principle of such a procedure within a practice-based convenience sample of BPD patients. We note, however, that only about half of the patients that were planned to follow during the DBT treatment did not volunteer or dropped out of this study. This observation is important for assessing the feasibility of employing these procedures broadly in clinical practice. Future qualitative, implementation research on facilitators and barriers to these procedures is warranted.

Further limitations of our study deserve special attention: First, our main result is based on a small sample size. Although we assessed the robustness of the effect extensively, for example, by cross-validation (leave-one-participant-out procedure) and by permutation-based analyses (SnPM), replication of our data is needed. Second, because we did not include a patient control group, we cannot assess whether the amygdala signal is a general predictor of positive change in symptomatology or whether it specifically moderates treatment outcomes. Third, our paradigm was insensitive to appetitive PIT (Supplementary Material) and therefore we cannot make any claims on the valence specificity of the presented results.

**FIGURE 5:**
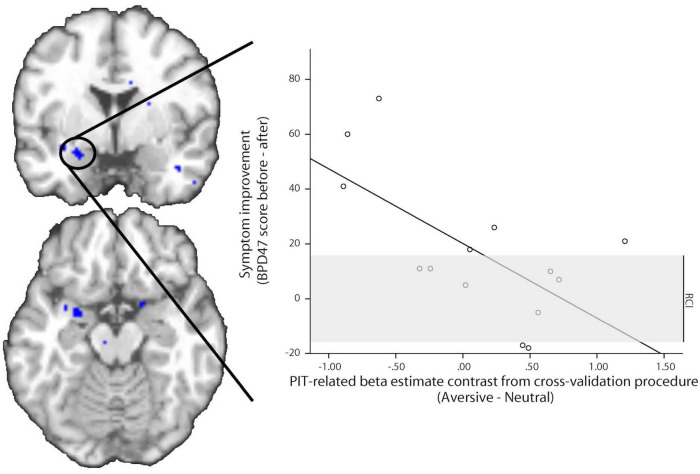
Association between amygdala BOLD signal change and symptom improvement. Pre-treatment PIT-related BOLD signal in the left amygdala predicts symptom improvement 1 year later. Images are displayed at a statistical threshold of *p* < 0.001 uncorrected. The scatter plot shows the PIT-related beta estimate contrast for aversive minus neutral CS trials before treatment in relation to symptom improvement, derived from a leave-one-participant-out cross-validation procedure. The regression line is the ordinary least square line. The gray area depicts the reliable change index (RCI) range; the changes outside this area are regarded as reliable (based on Jacobson and Truax, 1991).

Fourth, we set out to include all the patients who were offered DBT during the inclusion period of this study. In this setting, all BPD patients were female, which thus precludes conclusions about male BPD patients. Moreover, the inclusion resulted in a ‘real-life’ BPD patient group with the majority of patients being on psychotropic medication and having multiple comorbidities. This choice of patient selection was at the expense of internal validity [e.g., a recent meta-analysis shows that medicated compared with non-medicated patients with BPD show blunted amygdala responses (Schulze et al., 2016)], which we deliberately traded off against enhanced external validity (Hoertel et al., 2015). The majority of patients in normal clinical practice with BPD have multiple comorbidities and, although discouraged in many guidelines, take psychotropic medications, such as selective serotonin inhibitors. We acknowledge that we cannot exclude the possibility that differences in medication use contribute to the observed effect. With the low sample size and the diverse medication regimens of the included patients, we have no means to address this quantitively. To provide as much insight as possible, we report medication use in the Supplementary materials for each patient (Supplementary Table 1) and also added a graph, similar to Figure 5, showing bilateral amygdala signals for those with and without medication use (Supplementary Figure 1). Choosing such a sample is in line with our ultimate aim to find useful biobehavioral markers to predict and optimize treatment success in real-life clinical practice.

## Data availability statement

The raw data supporting the conclusions of this article will be made available by the authors, without undue reservation.

## Ethics statement

The studies involving human participants were reviewed and approved by METC Oost-Nederland. The patients/participants provided their written informed consent to participate in this study.

## Author contributions

DG, QH, TV, and RC contributed to conception and design of the study. DG collected the data and performed the statistical analysis under supervision of RC. DG and RC wrote the first draft of the manuscript. All authors contributed to manuscript revision, read, and approved the submitted version.
