# Supplementary material for: Amygdala response predicts clinical symptom reduction in patients with borderline personality disorder: A pilot fMRI study
Source: Front Behav Neurosci. 2022 Aug 30;16:938403. doi: 10.3389/fnbeh.2022.938403 (PMC9468714; doi:10.3389/fnbeh.2022.938403)
Supplement: Supplementary file 1 [file Data_Sheet_1.docx]

Supplementary Material

1. **Supplementary materials and methods**
   1. **Additional details on subject inclusion**

Exclusion criteria for all participants were severe somatic and neurological illness, mental retardation, severe hearing and visual disabilities, illicit drug use in the week before the experiment, alcohol or benzodiazepine use within 24-hours of the experiment and smoking 3 hours before scanning: Twenty-three patients volunteered. One patient could not participate due to metal in his spine (the only male in the sample), and one patient failed to attend the screening session. Twenty-one patients attended the intake session. For subsequent assessments, two patients were excluded because they did not meet the DSM-IV criteria of BPD. One patient reported not wanting to participate out of anxiety for the aversive juice. Two patients did not show up on the first scan session and we were unable to subsequently establish contact. During the first scan session one patient experienced a panic attack. The final dataset included 15 patients. Furthermore, one subject did not attend the one year follow-up session. Four of the other 14 patients who attended this session reported not having completed the full year of therapy.

- 1. **Image acquisition**

ME-EPI sequence details: 38 axial-oblique slices, repetition time, 2.250s; echo-times: 9.7, 20.3, 31, 41 and 52ms; in plane resolution, 3.5x3.5mm; slice thickness, 2.5mm; distance factor 0.17; flip angle, 96. Visual stimuli were projected on a screen and viewed through a mirror attached to the head coil. In addition, a high-resolution T1-weighted magnetization-prepared rapid-acquisition gradient echo anatomical scan was obtained from each subject (192 sagittal slices; repetition time, 2.3s; echo time, 3.03ms; voxel size 1.0 x 1.0 x 1.0 mm; field of view 256 mm).

- 1. **Supplementary analyses**
     1. **Instrumental and Pavlovian training**

The behavioral data were analyzed using the statistic software SPSS 16.0. First, we assessed whether subjects learnt the instrumental training task. The proportion of correct responses was calculated for the first ten and last ten trials separately for each of the four trial types. Performance (p(correct), not normally distributed) was compared between groups and between the beginning and the end of instrumental training by means of Wilcoxon Signed Rank and Mann Whitney U tests respectively. To assess differences in Pavlovian conditioning between the groups we compared performance on the Pavlovian query trials (p(correct), not normally distributed) by means of Mann Whitney U tests. In addition, liking ratings (not normally distributed) of the CSs before and after the experiment were analyzed using Mann Whitney U and Wilcoxon Signed Rank tests.

- - 1. **Supplementary neuroimaging analyses**

Secondary to our main analysis we fully explored the 2x2x2 rmANOVA using the parametric PIT regressors with Action Context (approach/withdrawal) and Valence (neutral/aversive) and Group(healthy controls/borderline personality disorder).

Moreover, we additionally assessed the relation between CS dependent BOLD signal change during the PIT stage and average behavioural PIT scores on a subject by subject basis. Therefore, in addition to the main effect of Valence [(approach neutral+withdrawal neutral) - (approach aversive+withdrawal aversive)] (see main paper), we calculated an interaction between Valence and Action Context [(approach neutral-approach aversive)-(withdrawal neutral-withdrawal aversive)]. The resulting SPMs for each contrast were then used in a two-sample t-test at the group-level with behavioural aversive PIT-effects (p(go) and average number of button presses) as a covariate for each group separately enabling comparison between groups. These analyses revealed additional regions in which individual differences in BOLD responses were linearly associated with individual differences in behavioural PIT in terms of choice and vigour respectively across and between groups.

- - - 1. **Additional generalized psychophysiological interaction (gPPI) analyses**

We used a generalized form of context-dependent psychophysiological interaction (gPPI, <http://brainmap.wisc.edu/PPI>, [McLaren et al., 2012](http://www.ncbi.nlm.nih.gov/pmc/articles/PMC4053644/#bb0510)). To compose the physiological variable, the extracted mean time series of the BOLD signal from the vmPFC blob (Figure 3, main paper) were temporally filtered, mean corrected, and de-convolved to generate the time series of the neural signal for the vmPFC for each individual subject. These time series of neural signal were then multiplied by the onset times of the trials with different CS Valence and separately with the parametric PIT-regressor (vector consisting of total number of button presses per trial per Action Context and CS Valence). The products were then re-convolved with the canonical HRF to obtain the interaction term or PPI variable.

Next, these regressors were added to the first level GLM described in the main paper.

The parameter estimates of the PIT-related PPI regressors (those based on the product of the seed time series and parametric PIT-regressor) quantify the relation with trial-by-trial instrumental action (i.e. number of button presses) and functional connectivity between the seed region (i.e. vmPFC) and other regions. Contrasting the PIT-related PPI regressors between CS Valence (and/or Action Context) reveals regions which functional connectivity with the vmPFC is differentially related to instrumental action as a function of CS Valence, thus representing PIT-related functional connectivity.

Based on Geurts et al. (2013) we expected that functional connectivity between the vmPFC and caudate nucleus would be dependent on CS Valence during withdrawal. Therefore, we submitted the beta-estimates resulting from the contrast [neutral|withdrawal] – [aversive|withdrawal] to a two sample t-test. We also tested, following the results of Geurts et al. (2013) whether the beta-estimates resulting from the contrast [neutral|approach] – [aversive|approach] were related to behavioral aversive PIT in terms of button presses [BP|approach&neutral - BP|approach&aversive].

- 1. **Supplementary Results**
     1. **Instrumental conditioning**

Overall subjects learned to make correct choices during the instrumental learning stage indicated by an increasing number of correct responses over time (p(correct|Time Bin1) vs p(correct|Time Bin2): Related Samples Wilcoxon Signed Rank Test: p<.001) (Figure S1). There was no difference in learning between the groups, not across Action Contexts and not for one of Action Contexts separately (Mann Whitney U test: all p>.616). Furthermore, during the PIT stage performance was the same for the groups across both Action Contexts and for each Context separately (Mann Whitney U test: all p>.616).

- - 1. **Pavlovian conditioning**

Mann-Whitney U tests showed there were no differences between the groups in how they rated the aversive and appetitive juice before and after conditioning (for all 4 comparisons p>.381). There was also no difference between the groups in pre to post changes in rating for the different juices (p>.669). Furthermore, there was no difference in performance on the Pavlovian query trials (Mann-Whitney U test: mean proportion correct over blocks in BPD: 94%; SEM: 2.2; range: 75-100%; HC: 93%; SEM: 3.4; range: 50-100%, p=.800). VAS ratings for the Pavlovian CSs (Figure S1) showed that the aversive CS became aversive to the participants (Wilcoxon Signed Rank Test: p=.001, one-tailed) and that the neutral and appetitive CSs did not change (Wilcoxon Signed Rank Test: p>.181, one-tailed; Figure S1). However, after conditioning the aversive CS did not differ significantly on VAS rating from the neutral CS, but did differ from the appetitive CS (Mann-Whitney U test: p=.031, one-tailed) and the appetitive CS was judged more appetitive than the neutral CS (Wilcoxon Signed Rank Test: p=.027, one-tailed). None of the VAS ratings for the Pavlovian CSs or their changes from before to after conditioning differed between the groups (All Mann-Whithney U tests: p>.381).

- - 1. **Pavlovian to instrumental transfer stage**
       1. *Vigour*

Analysis of vigour (i.e. number of button presses) revealed a main effect of Action Context, due to more vigorous responding during approach than withdrawal (Table S2, F1;29=33.7, p<.001). There were no significant differences between the groups in terms of the vigour of responding.

- - - 1. *Appetitive PIT*

As in previous studies with this task, we did not observe significant appetitive PIT (neutral vs. appetitive). The rmANOVA with Action Context(approach/withdrawal) and CS Valence(neutral/appetitive) as within subject factors and Group(HC/BPD) as between subject factor did not reveal any significant PIT-effects either with choice (p(go), all F<2.4, all p>.05) or with vigour (number of button presses, all F<3.18, all p>.05) as dependent variable.

- - 1. **Supplementary neuroimaging results**

In addition to the Action Context specific signal in the vmPFC (see main paper), we also observed action-specific signal in the precuneus, lingual and middle occipital gyrus (Table S3).

Significant brain-behaviour correlations were observed in the left amygdala (Table S3). Subjects showing increased Action Context specific aversive PIT in terms of behavior also showed Action Context specific responses of the amygdala to the aversive compared to the neutral CS. Thus, subjects who showed increased aversive inhibition of approach actions together with increased aversive activation of withdrawal actions also showed this aversive CS induced pattern with respect to amydgalar BOLD response.

No significant findings were revealed by the additional analysis of functional connectivity between the vmPFC and caudate nucleus.

- 1. **Supplementary Tables**

Table S1 *Medication use and MINI-plus classifications before treatment*

| SJ# | Psychoactive Medication (prescribed and non-prescribed) | SCID-II BPD #items | MINI -plus  Classifications @baseline | Therapy Completed |
| --- | --- | --- | --- | --- |
| 1 | Topiramate | 6 |  | Yes |
| 2 | Citalopram | 7 | MDD, PTSD, alcohol dependence and abuse, boulemia nervosa, hypochondria | Yes |
| 3 | Citalopram | 8 |  | No |
| 4 | Ventolin. melatonin | 6 | Past abuse and dependence of marihuana and XTC | No |
| 5 | Lamotrigine, trazolan, ezomeprazol, zeracette, diazepam | 7 | MDD | No |
| 6 | Diazepam, oxazepam | 8 | MDD, agorafobia | Yes |
| 7 | - | 8 | Agorafobia, PTSD, alcohol dependence and abuse, GAD, ADHD | Yes |
| 8 | - | 8 |  | No |
| 9 | Topiramate | 6 | Bipolar II disorder | Yes |
| 10 | Paroxetine | 8 | Abuse of XTC and GHB, PMS | Yes |
| 11 | - | 8 | MDD, panic disorder, agoraphobia, social fobia | Yes |
| 12 | - | 9 | MDD, PTSD, | Yes |
| 13 | Jasmin OAC | 6 | Dysthymia, simple fobia, boulimea nervosa | Yes |
| 14 | Simbicort  Oxycontin | 9 | MDD, agorafobia, social fobia, PTSD, ADHD | Yes |
| 15 | Temazepam20 mg ante noctem | 8 | MDD, boulimea nervosa | No |

MDD, major depressive disorder; PTSD, post traumatic stress disorder; ADHD, attention deficit and hyperactivity disorder; PMS, post-menstrual syndrome; GAD: general anxiety disorder

**Table S2** *Presented are the average number of button presses for the healthy control (HC) and borderline personality disorder (BPD) group as a function of Action Context (approach/withdrawal) and CS Valence (appetitive/neutral/aversive) during the Pavlovian-instrumental transfer stage (standard deviation)).*

|  | Action Context | | | |  |
| --- | --- | --- | --- | --- | --- |
|  | Approach | | Withdrawal | | |
|  | HC | BPD | HC | BPD | |
| Appetitive | 7.1(1.6) | 7.8(1.3) | 8.1(1.3) | 8.4(1.2) | |
| Neutral | 6.8(1.6) | 7.7(1.2) | 8.0(1.5) | 8.3(1.3) | |
| Aversive | 6.9(1.6) | 7.5(1.8) | 8.1(1.2) | 8.6(1.2) | |
|  |  |  |  |  | |

**Table S3** *Supplementary fMRI results*

| **Location** | **k** | **X** | **Y** | **Z** | **Z-value** | **P-value**  **FWE- cor** | |
| --- | --- | --- | --- | --- | --- | --- | --- |
| **Full Factorial: Group x Action Context x Valence (PIT-regressor)** | | | | | | | |
| **F-test: Main effect of Action Context**: | | | | | | | |
| Lingual and Calcarine (Bil) | 3547 | -12 | -76 | 2 | Inf | | P_WB_<.001 |
| Mid Occipital (L) | 147 | -42 | -68 | 0 | 5.6 | | P_WB_<.001 |
| Supramarginal gyrus (L) | 341 | -60 | -22 | 38 | 5.95 | | P_WB_<.001 |
| Mid Occiptal (R) | 111 | 36 | -82 | 8 | 4.96 | | P_WB_=.025 |
| Precentral (L) | 78 | -22 | -12 | 54 | 4.81 | | P_WB_=.048 |
|  |  |  |  |  |  | |  |
| **F-test: Interaction Group x Action Context x Valence** | | | | | | | |
| White matter near inferior frontal gyrus | 60 | 32 | -2 | 26 | 4.88 | | P_WB_=.036 |
|  | | | | | | | |
| **2 sample T-test: Main regressor: Action Context specific aversive PIT contrast + covariate of interest: behavioural action specific aversive PIT (button presses)**  **[Neu\|Approach]-[Ave\|Appraoch] – ([Neu\|Withdrawal]-[Ave\|Withdrawal])** | | | | | | | |
| T-test: main positive effect of covariate (across groups): | | | | | | | |
| Amygdala left | 45 | -30 | 0 | -18 | 4.86 | | P_sv_=.003 |

- 1. **Supplementary Figure**


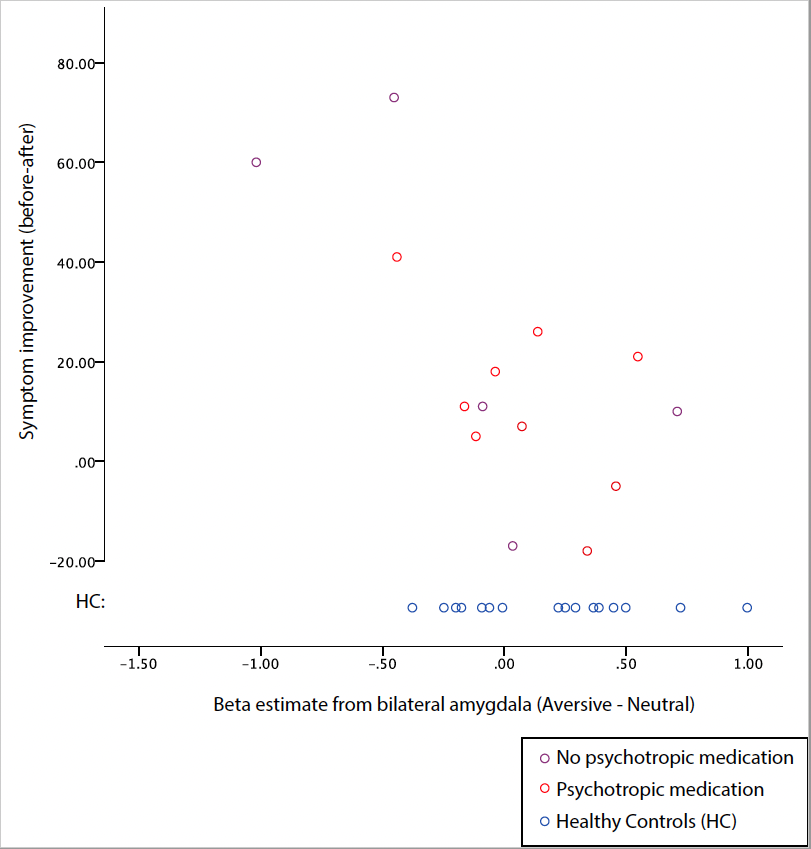


**Figure S1.** Association between amygdala BOLD signal change and symptom improvement with additional information about healthy controls (HC) and medication use. The scatter plot shows the PIT-related beta estimate contrast for aversive minus neutral CS trials before treatment, extracted from the bilateral amygdala ROI, in relation to symptom improvement for BPD patients. HC did not receive treatment and were only assessed once, therefore they are depicted separately.
